# Bridging the cancer divide: Policy and structural solutions for equity in the United States

**DOI:** 10.3322/caac.70054

**Published:** 2025-12-16

**Authors:** Mariana Chavez‐MacGregor, Inimfon Jackson

**Affiliations:** ^1^ Department of Health Services Research The University of Texas MD Anderson Cancer Center Houston Texas USA; ^2^ Department of Breast Medical Oncology Division of Cancer Medicine The University of Texas MD Anderson Cancer Center Houston Texas USA; ^3^ Division of Cancer Medicine The University of Texas MD Anderson Cancer Center Houston Texas USA

“Of all the forms of inequality, injustice in health is the most shocking and the most inhuman because it often results in physical death.”^1^

–Martin Luther King Jr.

Over the last few decades, there has been significant progress in the fight against cancer. In the United States between 1991 and 2022, cancer‐related mortality decreased, with an estimated 4.5 million fewer deaths.^2^ This is because of numerous factors, including reductions in smoking, early detection for cancers with available screening, and improved therapies in the management of both localized and metastatic cancer.^2^ Despite such improvements, the American Cancer Society's (ACS) Report on the Status of Cancer Disparities by Islami and colleagues that accompanies this editorial, sheds light on the persistent and substantial disparities across the cancer care continuum, including risk factors, incidence, stage at diagnosis, receipt of care, survival, and mortality for many cancers.^3^ The report evaluates not only the effect of race and ethnicity but also educational status, geographic location, sex, and social determinants of health. In a nation where some of the world’s most advanced treatments and medical technologies are available, it is tragic that not all individuals benefit equally from these innovations. These persistent cancer disparities reflect complex interactions between race and ethnicity, socioeconomic status, and geography.^4^ Systemic inequities and policies that have not satisfactorily addressed the most vulnerable have widened the gap between the rich and the poor, disproportionately affecting racial and ethnically minoritized individuals.^5^

According to the 2025 ACS report, the incidence of cancer is higher among Black and American Indian/Alaska Native men and women compared with White individuals across multiple cancers and, notably, these populations also have the highest cancer‐related mortality rates.^3^ Strategies to improve cancer outcomes have led to a modest narrowing of these gaps over time; however, Black–White mortality disparities persist.^6^ Although cancer disparities are frequently described according to race and ethnicity, to better understand the implications of this report, it is crucial to detangle the concept of race, which nonetheless remains a strong predictor of higher cancer incidence and worse survival.^7^ The statistics presented in the ACS report highlight that, beyond race and ethnicity, factors like inequitable access to care, poverty, social determinants of health, and other structural factors are key drivers of disparities and associated poor outcomes, rather than biologic differences alone.

Social determinants of health unequally affect racial and ethnically minoritized individuals, compromising their opportunities to have health care outcomes comparable to those of White populations. Black, American Indian/Alaska Native, and Hispanic individuals are more likely to be poor, less educated, lack health insurance, and experience food and house insecurity compared with their White counterparts. For example, in 2024, about 21.4% of American Indian/Alaska Native, 16% of Black, and 15.7% of Hispanic individuals aged 18–64 years had incomes below the poverty level compared with 8.7% of Asian and 7.7% of White individuals.^3^ These inequities are associated with decreased access to cancer prevention services, diagnosis of cancer at later stages, treatment delays, and worse survival outcomes.^3^, ^4^ In addition, structural factors like racial residential segregation, economic exclusion, discriminatory policies, and limited health care infrastructure reinforce these disparities, creating barriers to quality health care. The combination of all these factors perpetuates a cycle of disadvantage that sustains the poor health outcomes and inequities over time in minoritized populations.^8^, ^9^

Given these structural inequities and barriers, a call for action is imperative. For example, improving access to affordable and high‐quality health insurance is a way to achieve health equity. The 2025 ACS report indicates that uninsured adults aged 18–64 years are significantly more likely to delay or not receive necessary medical care because of cost, leading to missed or late screenings for cancers such as colorectal and female breast cancers.^3^ These findings highlight the urgent need to protect and expand access to affordable health insurance for disadvantaged populations. Although Medicare protects most adults older than 65 years by providing medical coverage, younger individuals, particularly those with lower income and educational levels and those residing in nonmetropolitan areas, remain at risk.^3^ Studies have demonstrated that Medicaid expansion increased insurance coverage, improved cancer screening, and reduced mortality rates among low‐income adults,^10^, ^11^, ^12^ strongly suggesting that policy changes can lead to a direct improvement of cancer outcomes and favor a path to improved health equity. Notably, as highlighted in the ACS report, the congressional districts with the highest overall cancer mortality rates include the South and East North‐Central division of the Midwest,^3^ and correspond in great part with districts in states that did not expand Medicaid under the Affordable Care Act of 2010. Given the evidence, one could argue that we must sustain and seek to expand Medicaid and marketplace subsidies and incentivize the nonexpansion states to do the same to ensure that all individuals have access to cancer prevention services and timely cancer care.

When analyzed by urbanicity, the ACS report demonstrates that nonmetropolitan areas have higher cancer mortality rates compared with large metropolitan areas, with rates increasing by 23% in men and 18% in women from 2019 to 2023.^3^ The largest differences were noted in lung cancer (47% higher in men and 40% higher in women), cervical cancer (36% higher), and colorectal cancer (28% higher).^3^ These are cancers that either are linked to modifiable risk factors or can be diagnosed at early stages by screening. Telehealth programs, mailed at‐home screening test kits, and the use of mobile screening units have been successful in increasing access to cancer screening and follow‐up care.^13^, ^14^, ^15^ Early cancer detection could be further improved by sponsoring multifaceted screening programs to reduce the proportion of preventable cancers diagnosed at later stages in rural communities. In addition, telehealth should be easily accessible to both physicians and their patients to facilitate cancer prevention visits and testing, particularly where transportation to appointments is challenging.

Cancer disparities also affect the receipt of cancer‐directed treatment. Individuals who have lower income, have no insurance coverage, or who reside in rural areas are less likely to receive guideline‐concordant care.^3^ Reports across multiple cancer types support that such groups of individuals also are more likely to experience financial toxicity, fragmentation of care, and limited access to specialty providers, particularly in rural areas.^16^, ^17^ They are also more likely to experience treatment delays and, consequently, worse outcomes. Patients with low socioeconomic status or who receive treatment at facilities where predominantly individuals of low socioeconomic status reside, are less likely to receive standard treatments. Furthermore, those living in rural areas face additional barriers like needing to travel long distances to receive care, incurring significant transportation expenses. Consequently, they are at increased risk to experience financial toxicity and also nonadherence, all likely contributing to higher mortality rates.^18^ Policy and institutional strategies are needed to address both patient‐level and facility‐level barriers to ensure equitable access to timely, high‐quality, and guideline‐concordant cancer care for all.

In addition to disparities in treatment delivery, underrepresentation of minoritized patients in clinical trials remains a major challenge to equitable cancer care. Research aimed at understanding the barriers to clinical trial participation suggest that these barriers exist at multiple levels,^19^ including structural, institutional, provider level, patient level and study level barriers. Decentralizing clinical trials has shown to significantly improve access to clinical trials for patients in community settings^20^ and we argue that it should be prioritized. Federally funded research, specifically the National Clinical Trials Network and the National Cancer Institute Community Oncology Research Program, are two cornerstone programs of the US National Cancer Institute that, together, make cancer clinical trials more accessible, equitable, and representative across the country. These programs connect academic centers, community hospitals, and private practices into a big network conducting clinical trials. In addition, the National Cancer Institute Community Oncology Research Program supports studies in cancer prevention, symptom management, and survivorship in addition to cancer care delivery research. It has been demonstrated that both programs are powerful tools for bringing clinical trials to community practices across the country. Their existence is key to ensuring clinical trial access for all but is particularly important for minoritized groups and those living in rural areas, who otherwise have limited opportunities to participate in potentially life‐saving research.

The ACS report by Islami et al. highlights important disparities that affect not only vulnerable and marginalized populations but also entire communities, the society as a whole, and the nation. To improve health equity, major structural changes aimed at addressing poverty and access to health care are needed. These include, among others, expanding Medicaid eligibility; increasing funding for cancer screening programs, such as the National Breast and Cervical Cancer Early Detection Program; and supporting access to multicancer early detection tests and biomarker testing. Patient navigation and community‐based participatory research have proven to be effective in improving patient‐centered outcomes in minoritized groups, but funding these programs remains a challenge.^18^ Efforts are also needed to increase diversity in the cancer care and research workforce to identify and address disparities. Sustained investment in research addressing disparities that include culturally tailored interventions are required to ensure that all populations derive benefit from the extraordinary advances made in cancer care. Only coordinated policy action and the implementation of proven strategies will help us achieve meaningful, population‐level reductions in cancer disparities so we can improve cancer outcomes for all.

## CONFLICT OF INTEREST STATEMENT

Mariana Chavez‐MacGregor reports personal/consulting fees from AstraZeneca, Eli Lilly and Company, Genentech, Genomic Health, Gilead Sciences Inc., Novartis, and Pfizer; support for other professional activities from Genentech, Novartis, and Merck; and data and safety monitoring for AstraZeneca and Genentech outside the submitted work. Inimfon Jackson disclosed no conflicts of interest.
